# Evaluation of the surface water quality using global water quality index (WQI) models: perspective of river water pollution

**DOI:** 10.1038/s41598-023-47137-1

**Published:** 2023-11-22

**Authors:** Md. Habibur Rahman Bejoy Khan, Amimul Ahsan, M. Imteaz, Md. Shafiquzzaman, Nadhir Al-Ansari

**Affiliations:** 1 Department of Civil and Environmental Engineering, Islamic University of Technology, Gazipur, Bangladesh; 2 Department of Civil and Construction Engineering, Swinburne University of Technology, Melbourne, Australia; 3 Department of Civil Engineering, College of Engineering, Qassim University, 51452 Buraidah, Saudi Arabia; 4 Civil, Environmental and Natural Resources Engineering, Lulea University of Technology, 971 87 Lulea, Sweden

**Keywords:** Freshwater ecology, Freshwater ecology, Environmental impact, Hydrology

## Abstract

Rapid industrialization, urbanization, global warming, and climate change are compromising surface water quality across the globe. Consequently, water conservation is essential for both environmental sustainability and human survival. This study assesses the water quality of the Jamuna River in Bangladesh at five distinct sites during wet and dry seasons. It employs six global water quality indices (WQIs) and contrasts the results with Bangladesh's Environmental Quality Standard (EQS) and the Department of Environment (DoE) criteria. The WQI models used are the Weighted Arithmetic WQI (WAWQI), British Columbia WQI (BCWQI), Canadian Council of Ministers of the Environment WQI (CWQI), Assigned WQI (AWQI), Malaysian WQI (MWQI), and Oregon WQI (OWQI). Fifteen physicochemical parameters were analyzed according to each WQI model's guidelines. The findings reveal that most parameters surpass the standard permissible values. The WQI model results indicate that the average water quality across the five sites falls into the lowest category. A comparison of the WQI models suggests potential correlations between WAWQI and AWQI, as well as between MWQI and OWQI. The straightforward presentation of the WQI models indicates that while the river water requires treatment for household and drinking use, it remains suitable for irrigation. The decline in water quality is likely attributable to human activities, urbanization, municipal waste disposal, and industrial effluents. Authorities must prioritize regular monitoring and assessment of water quality to address the identified challenges. Restoring the water to an acceptable standard will become increasingly difficult without proactive measures.

## Introduction

Water covers 70% of the Earth's surface and is vital for human survival due to its diverse applications, including irrigation, drinking, cooking, cleaning, and various industrial activities^1,2^. This essential resource can be sourced from both surface and subsurface reservoirs^3^. Generally, groundwater may have lower levels of organic pollutants compared to surface water bodies such as rivers, lakes, and ponds. This is because groundwater undergoes natural filtration as it percolates through the soil and rocks, reducing impurities. Consequently, groundwater treatment is often more straightforward and requires fewer steps than surface water treatment^4,5^. However, river water quality has seen a decline in recent years, primarily due to human activities such as domestic waste disposal, agriculture, urbanization, and industrialization, although natural factors also play a role^6–8^. Seasonal rainfall further exacerbates river pollution by facilitating surface runoff, which often carries industrial and municipal wastewater^9^. The WHO^10^ reported that approximately 159 million people globally rely on unsafe surface water sources, which pose significant health risks. This challenge is particularly pronounced in developing countries like Bangladesh, where many rural inhabitants depend on surface or groundwater for their daily needs^11^. Water quality degradation, influenced by rising temperatures, trace elements, and pollutants like phosphorus and nitrogen, is becoming a pressing concern worldwide^12,13^. Additionally, the contamination of water by nitrate and fluoride has surged globally over the past two decades, posing severe health implications^14^. Monitoring river water quality is crucial for both ecosystem preservation and human health protection. Given the vulnerability of river water, frequent assessments are essential to devise sustainable management strategies, especially considering the increasing pollution sources from human activities and urbanization^15,16^. In summary, prioritizing regular evaluation and monitoring of river water quality is paramount for global water resource conservation^17^.

Various techniques and models have been devised to evaluate the diverse water quality parameters of rivers affected by different pollutants, aiming to present the results in a straightforward manner. A challenge arises due to the myriad factors influencing water quality and the vast array of metrics used to define water quality across different water bodies^18^. Moreover, evaluating a large set of samples, each with multiple parameters is daunting^19^. To simplify this process, the Water Quality Index (WQI) was introduced and has since gained global acceptance. Horton first proposed the WQI in 1965, using ten water parameters to assess river water quality^20^. Since then, many researchers worldwide developed their WQIs, including notable indices like the National Sanitation Foundation WQI and Weighted Arithmetic WQI (WAWQI) by Brown et al.^21^, British Columbia WQI (BCWQI) by Zandbergen and Hall^22^, Canadian Council of Ministers of the Environment WQI (CWQI) by CCME^23^, Assigned WQI (AWQI) by Alobaidy et al.^24^, Malaysian WQI (MWQI) by DOE^25^, and Oregon WQI (OWQI) by Cude^26^. The WQI calculation methods have significantly enhanced water quality determination, monitoring, and evaluation in recent times. Thus, it's crucial to recognize the value these methods bring to water quality management and regulatory decisions. Numerous studies have employed the WQI to assess surface water quality^27–38^. For instance, Dimri et al.^28^ highlighted that the Ganga River in India faces pollution from various sources, including household waste, industrial effluents, fertilizer runoff, religious activities, and natural weathering. They emphasized the need for monitoring and policy implementation to curb this pollution. Similarly, a study on Turkey's Karasu River identified pollution sources as natural, seasonal, phytoplankton-related, and anthropogenic^38^. Another investigation into Nigeria's Okulu River revealed its water quality as unsuitable for drinking or irrigation^29^. These studies underscore the importance of utilizing the WQI to devise effective mitigation strategies, pinpointing the primary culprits behind river pollution.

Bangladesh, being a riverine country, relies heavily on its rivers. These water bodies play a pivotal role in the ecosystem, influencing various environmental aspects and significantly shaping the lives of millions of its residents^39^. The southern stretch of the Brahmaputra River in Bangladesh is known as the Jamuna River, which flows from the north to the south. Each year, erosion claims thousands of hectares of mainland floodplain along this river and its islands, displacing numerous individuals from their homes and means of livelihood^40^. Seasonal rainfall is a primary contributor to this riverbed erosion. Moreover, climate change and global warming are exacerbating the erosion, accretion, and migration of riverbanks. These phenomena alter rainfall patterns, leading to floods and shifts in discharge patterns and water flows^41,42^. While there are limited studies on the WQI of Bangladesh's rivers, no WQI study have been conducted on the Jamuna River, a crucial water body with significant socio-economic, ecological, and geographical implications. Studying its water quality not only addresses a regional knowledge gap but also provides insights that can benefit river systems facing similar challenges worldwide. One notable study on the Surma River using WQI revealed a decline in water quality due to the influx of municipal pollutants^30^. Another investigation by Islam et al. (2011) on the Titas River determined that its water is suitable for recreational, irrigation, and pisciculture purposes but requires treatment for potable use^43^. Given these findings, it is imperative for the government to take action in implementing these necessary management and monitoring programs to safeguard river water quality for both the ecosystem and public health.

This study aims to assess the water quality of the Jamuna River at five distinct locations during both the wet and dry seasons. This assessment utilizes six global WQIs, namely WAWQI, BCWQI, CWQI, AWQI, MWQI, and OWQI. These indices are selected based on their relevance to the region, comprehensive parameter inclusion, global applicability, and variation in assessment methodology to ensure a well-rounded and thorough evaluation of the Jamuna River's water quality. This is the first time to apply a number of WQIs for the same river to check whether the models can predict the same or similar results. This in-depth comparison to determine the consistency/similarity of these models' predictions is required as it is not deeply explored yet in the literature. Consequently, the suitability of the stated models can be judged for the Jamuna River water quality predictions. This study also delves into potential reasons for the deterioration of the Jamuna River's water quality and calls upon authorities to take remedial actions. The outcome of this pioneering investigation on WQI evaluation highlights the urgent need of effective management strategies to curb river water pollution.

## Methodology

### Data collection

Data on the Jamuna River was sourced from Uddin et al.^44^ and DoE^45^, as presented in Table A1 (in Supplementary Materials). The river's physicochemical parameters—including temperature, EC (Electrical Conductivity), pH, BOD_5_ (Biological Oxygen Demand), TDS (Total Dissolved Solids), TSS (Total Suspended Solids), TS (Total Solids), DO (Dissolved Oxygen), COD (Chemical Oxygen Demand), ammonia, nitrate, chloride, sulphate, and calcium—were analyzed for both the wet and dry seasons in Bangladesh using various laboratory guidelines. The DO (% of saturation) was determined by evaluating temperature, EC, and DO (mg/L) according to the FDEP (Florida Department of Environmental Protection^46^) 2013 guidelines. Additionally, since river turbidity is closely related to suspended solids, its value is determined using Eqs. (1) and (2), as proposed by Oliveira et al.^47^.

1 $$ {\text{TSS}}_{{{\text{wet}}}} = \, 0.{\text{86 turbidity }} + { 9}.{99} $$

2 $$ {\text{TSS}}_{{{\text{dry}}}} = \, 0.{\text{79 turbidity }} + { 4}.{36} $$

where TSS_wet_ is the wet season total suspended solids (mg/L), TSS_dry_ is the dry season total suspended solids (mg/L), and turbidity is measured in NTU (Nephelometric Turbidity Unit).

### Study area

**Figure 1:**
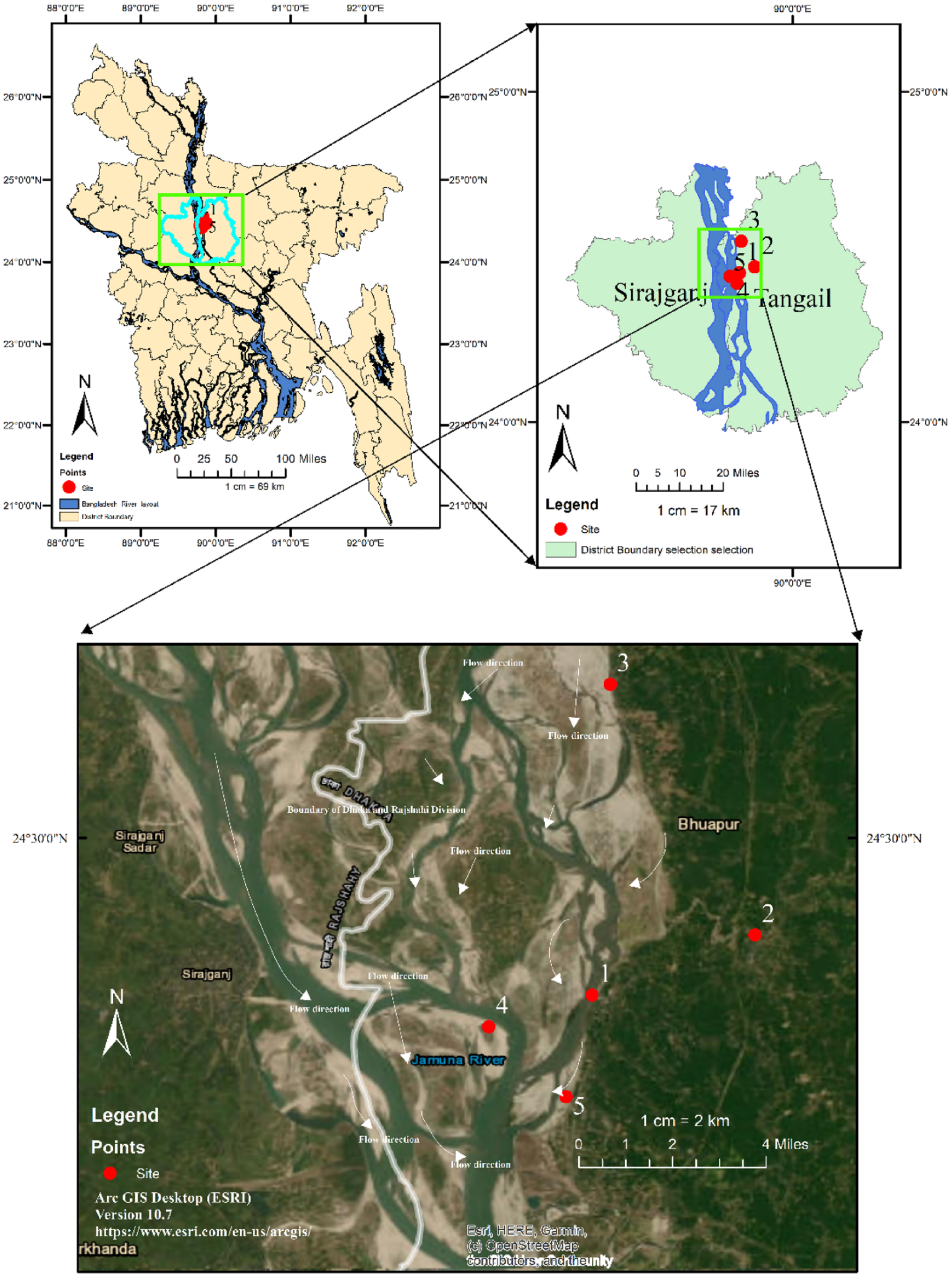
Study area with sample sites in Jamuna River.

Five study sites, labeled S1-S5, were examined along the Jamuna River in the Bhuapur Upazila of the Tangail district in Bangladesh, as depicted in Fig. 1. The Arc GIS Desktop software (ESRI^48^), version 10.7 was used to generate the Fig. 1 using the coordinates of the five sites illustrated in Uddin et al.^44^. The Jamuna River is a primary channel of the Brahmaputra, one of the world's major braided rivers, which flows from India into Bangladesh. The Jamuna spans a length of 240 km and reaches a maximum depth of 322 m. During the wet season (May–August), the river experiences its highest water levels and discharge, often leading to floods that inflict significant damage on the local population. The water level of the Jamuna fluctuates between 6 and 7 m across the dry and monsoon seasons^49^.

### Different WQI applications

Numerous WQIs have been formulated in recent years by different countries and researchers in order to assess the condition of surface and groundwater for different purposes like drinking, agriculture, and others^37^. The notable ones are Weighted Arithmetic WQI (WAWQI) by Brown et al.^21^, British Columbia WQI (BCWQI) by Zandbergen and Hall^22^, Canadian Council of Ministers of the Environment (CCME) WQI (CWQI) by CCME^23^, Assigned WQI (AWQI) by Alobaidy et al.^24^, Malaysian WQI (MWQI) by DOE^25^, and Oregon WQI (OWQI) by^26^. The methodology of the different WQIs is explained here.

### WAWQI

The WAWQI is a well-recognized index for determining the quality of water on the surface and ground, which helps give information on the water quality to the public and policymakers. The index is used to assess the impact of waste dumping on the immediate surface and groundwater bodies and is considered the most suitable index^21^. Fourteen parameters are used in generating the WAWQI of the Jamuna River, which are DO, pH, turbidity, TSS, TDS, ammonia, nitrate, sulphate, chloride, calcium, COD, BOD_5_, EC and temperature. The WQI equations of Oni and Fasakin^33^ and Brown et al.^21^ are applied as follows.

3 $$WQI= \frac{\sum_{i=1}^{n}{q}_{i}{w}_{i}}{\sum_{i=1}^{n}{w}_{i}}$$

where, q_i_ = quality rating of i^th^ water quality parameters and w_i_ = unit weight of i^th^ water quality parameters as follows.

4 $$\sum_{i=1}^{n}{w}_{i}=1$$

Furthermore, q_i_ correlates the value of the polluted water parameter with respect to the standard permissible value.

5 $${q}_{i}=(\frac{{v}_{i}-{v}_{io}}{{s}_{i}-{v}_{io}})\times 100$$

where, v_i_ = measured value of the i^th^ parameter, v_io_ = ideal value of the ith parameter, s_i_ = standard permissible value of the i^th^ parameter. In most cases, v_io_ = 0 except for DO and pH. For DO, v_io_ = 14.6 mg/L and for pH, v_io_ = 7. w_i_ is inversely proportional to the recommended standards as follows.

6 $${w}_{i}=\frac{k}{{s}_{i}}$$

where, $$k=\frac{1}{\sum_{i=1 }^{n}\frac{1}{{s}_{i}}}$$

The classification of WQI values in the weighted arithmetic index is shown in Table A2 (in Supplementary Materials) and w_i_ computations of the physicochemical parameters are illustrated in Table A3 (in Supplementary Materials), where the Bangladesh surface water quality guidelines are considered the standard permissible value for computation of the WAWQI.

### BCWQI

The British Ministry established the BCWQI in 1995 after conducting an assessment of more than a hundred water bodies in British Columbia to see how well they met water quality targets^22^. The calculation of BCWQI is done here with ten parameters, which are DO, pH, turbidity, TSS, TDS, ammonia, nitrate, sulphate, chloride and calcium. The equation of BCWQI is taken from Zandbergen and Hall^22^ and is as follows.

7 $$BCWQI=\sqrt{\frac{{F}_{1}^{2}+{F}_{2}^{2}+{(\frac{{F}_{3}}{3})}^{2}}{1.453}}$$

where, F_1_ represents the total number of objectives not met (as a percentage of all objectives checked), F_2_ represents the frequency with which objectives are not met (as a percentage of all instances of objectives being checked), and F_3_ represents the maximum deviation (as a percentage) for any one objective. The classification of WQI values for the BCWQI is given in Table A4 (in Supplementary Materials), where lower values indicate higher quality water.

### CWQI

The Canadian government developed the CWQI in 2001, following some of the aspects of BCWQI^50,51^ and CCME^23^. Thirteen parameters are utilized in calculating the CWQI here: DO, pH, turbidity, TSS, TDS, ammonia, nitrate, sulphate, chloride, calcium, COD, BOD_5_ and temperature. The formulation of CWQI done by CCME^23^ is as follows.

F_1_ (scope measure) indicates the percentage of parameters (out of a total number of parameters measured) that at least once during the time period under consideration do not meet the criteria.

8 $${F}_{1}=\left(\frac{Number\, of\, failed \,parameters}{Total\, number \,of \,parameters}\right)\times 100$$

F_2_ (frequency measure) indicates the percentage of tests that fail to meet the standard criteria.

9 $${F}_{2}=\left(\frac{Number \,of\, failed \,tests}{Total \,number \,of\, tests}\right)\times 100$$

F_3_ (amplitude measure) indicates how far test results deviate from the standard criteria and is calculated in three phases.

(a) The term "excursion" is used to describe the number of times a single concentration is above (or below, if the guideline is a minimum) the guideline. In cases when the measured value cannot go above the threshold:

10 $${excursion}_{i}=\left(\frac{Failed \,Test \,{Value}_{i}}{{Objective}_{j}}\right)-1$$

In cases when the test value cannot be below the threshold:

11 $${excursion}_{i}=\left(\frac{{Objective}_{j}}{Failed\, Test\, {Value}_{i}}\right)-1$$

(b) The overall deviation from standards and values is determined by adding all the excursions of all the tests and then dividing by the total number of tests (both those meeting guidelines and those not meeting guidelines). The following formula is used to calculate the normalized sum of excursions (nse) parameter:

12 $$nse=\frac{\sum_{i=1}^{n}{excursion}_{i}}{Total\, number \,of \,tests}$$

(c) An asymptotic function that adjusts the normalized sum of the excursions from recommendations (nse) to yield a range between 0 and 100 is then used to determine F_3_.

13 $${F}_{3}=(\frac{nse}{0.01\times nse+0.01})$$

The index can be computed by summing the three factors as vectors and applying Pythagoras' theorem. Sum of factor squares equals CCME WQI square. This method treats the index as a three-dimensional space with one axis for each factor. The index changes directly with all three elements in this model.

14 $$CWQI=100-(\frac{\sqrt{{F}_{1}^{2}+{F}_{2}^{2}+{F}_{3}^{2}}}{1.732})$$

The divisor 1.732 normalizes the results to a range between 0 and 100, where 0 is "worst" and 100 is "highest" water quality. The classification of CWQI values is shown in Table A5 (in Supplementary Materials).

### AWQI

The AWQI is formulated depending on the significance of various water parameters intended for use, and it is calculated using the standard water criteria. In this study, six parameters namely DO, pH, turbidity, nitrate, BOD_5_ and EC are used in assessing the AWQI. The computation of AWQI is done as follows:

(a) First, each of the parameters is assigned with a weight (AW_i_) ranging from 1 to 4 from the Alobaidy et al.^24^ study. The mean weight values are shown in Table A6 (in Supplementary Materials) along with the standard criteria. A relative weight of 1 was given the least importance, while a weight of 4 was given the most.

(b) In this step, the relative weight (RW) is found by diving the summation of assigned weight to each assigned weight and is calculated from the following equation:

15 $$RW=\frac{{AW}_{i}}{\sum_{i=1}^{n}{AW}_{i}}$$

where, RW = the relative weight, AW = the assigned weight, n = total number of parameters, which is illustrated in Table A6 (in Supplementary Materials).

(c) In this step, a quality rating scale (Q_i_) is assigned by dividing all the parameters by their respective standard permissible criteria except for pH and DO.

16 $${Q}_{i}=\left(\frac{{C}_{i}}{{S}_{i}}\right)\times 100$$

For pH and DO (Q_pH,DO_) is calculated by the following equation:

17 $${Q}_{pH,DO}=(\frac{{C}_{i}-{V}_{i}}{{S}_{i}-{V}_{i}})\times 100$$

where, Q_i_ = quality rating, C_i_ = measure water quality parameters, S_i_ = standard permissible criteria for water quality parameters., Vi = ideal value which is taken as 7 for pH and 14.6 for DO. Some conditions are used in Q_i_ and Q_pH,DO_ which are, Q_i_ = 0 when no pollutants in water and Q_i_ = 100 when the value of pollutants is equal to the standard permissible value. Hence, the greater the value of Q_i_ is, the more contaminated the water^52^.

(d) In the final step, the sub-indices (Sl_i_) are calculated for each parameter and then the summation of the total Sl_i_ is the WQI and the equations are as follows:

18 $${Sl}_{i}=RW\times {Q}_{i}$$

19 $$WQI=\sum_{i=1}^{n}{Sl}_{i}$$

The classification of the AWQI is done by the following Table A7 (in Supplementary Materials) where a lower value corresponds to better water quality.

### MWQI

The Malaysian Department of Environment developed an WQI in 1997 as illustrated by^53^ which used six water quality parameters (DO, BOD_5_, COD, TSS, ammonia, pH). All six parameters are used in this study to determine the value of WQI. The WQI formula used by Malaysia is as follows.

20 $$ {\text{WQI}} = 0.22 \times {\text{SI DO}} + 0.19 \times {\text{SI BOD}} + 0.15 \times {\text{SI AN}} + 0.12 \times {\text{SI pH}} + 0.16 \times {\text{SI COD}} + 0.16 \times {\text{SI SS}} $$

where SI denotes a sub-index value for each of the parameters, and the coefficients are the weighting elements based on the survey responses. The best fit equation for estimating the different sub-index is calculated by the DOE^25^ guideline. The classification of MWQI is shown in Table A8 (in Supplementary Materials) which illustrates the greater the value, the better is the quality of water.

### OWQI

The Oregon Department of Environmental Quality developed the OWQI in the 1970s in order to analyze and assess the different water quality categories and trends^54^. It was used for legally required water quality status assessment reports. It used the Delphi Technique to choose water quality characteristics and was modeled after the National Sanitation Foundation's WQI (NSFWQI). The water quality factors were categorized according to oxygen depletion, eutrophication, dissolved chemicals, and health threats. Six parameters are used in this study: DO, pH, summation of nitrate and ammonia, TS, BOD_5_ and temperature for the calculation of OWQI. Furthermore, the formula of TS in the Klamath Basin in Oregon state of the United States is considered in this study as it is required to choose a suitable basin to calculate TS. The formulation of OWQI results from the combination of NSFWQI and WAWQI, which results in an unweighted harmonic squared mean formula. The formula has been suggested as an improvement over NSFWQI and WAWQI by Dojlido et al.^55^. The equation is given below, as described by Cude^26^:

21 $$OWQI=\sqrt{\frac{n}{\sum_{i=1}^{n}\frac{1}{{SI}_{i}^{2}}}}$$

where, where n is the total number of subindices, and SI is subindex i of different parameters. The classification of OWQI is shown in Table A9 (in Supplementary Materials) which illustrates the smaller the value, the worse the quality of water.

## Results and discussion

### Seasonal variations (dry and wet seasons)

Table 1 presents a descriptive statistical analysis of five sites along the Jamuna River. The values for most physicochemical parameters significantly deviate from the standard permissible levels. During the wet season, the maximum values for pH, BOD5, and temperature (pH: 9.10, BOD5: 56 mg/L, and temperature: 36.10 °C) are higher than those in the dry season (pH: 8.8, BOD5: 40 mg/L, and temperature: 32.80 °C). Conversely, the maximum values for other physicochemical parameters in the wet season, such as turbidity (125.60 NTU), TSS (118.00 mg/L), TDS (110.00 mg/L), COD (9.80 mg/L), and ammonia (2.70 mg/L), are lower than their counterparts in the dry season (turbidity: 181.80 NTU, TSS: 148.00 mg/L, TDS: 130.60 mg/L, COD: 101.60 mg/L, ammonia: 12.00 mg/L, among others). This suggests that the water quality during the dry season is not suitable for use. In the dry season, a few potential factors are exacerbating water pollution of the Jamuna River such as reduced river flow, higher water withdrawal, and decreased groundwater recharge. It may collectively contribute to the degraded river water quality. Furthermore, even the average values of the physicochemical parameters fall below the standard permissible levels set by Bangladesh’s EQS^56^ and DoE^57^ guidelines. When comparing the water quality of the Jamuna River with other rivers in Bangladesh, such as the Surma River^30^, the Titas River^43^, and rivers near Dhaka like Turag-Buriganga and Balu-Sitalakhya^35^, it's evident that most rivers in Bangladesh face pollution challenges during the dry season.

**Table 1:** Physicochemical statistical analysis of the Jamuna River.

Parameter	Dry season					Wet season				
	Minimum	Maximum	Average	Standard Deviation	Variance	Minimum	Maximum	Average	Standard deviation	Variance
pH	8.40	8.80	8.60	± 0.98	0.96	8.60	9.10	8.90	± 1.17	1.37
Temperature (°C)	32.50	32.80	32.59	± 1.48	2.19	35.80	36.10	35.93	± 3.84	14.75
EC (µS/cm)	137.20	138.30	137.83	± 114.67	13,149.21	105.00	107.00	107.70	± 135.98	18,490.56
Turbidity (NTU)	118.5	181.8	159.04	± 21.83	476.70	68.6	125.6	93.26	± 20.24	409.69
TDS (mg/L)	129.30	130.60	130.04	± 24.72	611.08	109.00	110.00	109.48	± 39.26	1541.35
TSS (mg/L)	98	148	130	± 17.24	297.2	69	118	90.2	± 17.41	302.96
TS (mg/L)	228.6	278	259.98	± 17.04	290.55	179	227	199.68	± 17.22	296.86
DO (mg/L)	0.80	1.10	1.01	± 3.88	15.05	0.30	0.80	0.45	± 4.28	18.32
BOD_5_ (mg/L))	32.40	40.00	34.26	± 20.69	428.08	49.00	56.00	59.04	± 38.21	1460.00
COD (mg/L)	94.80	101.60	98.30	± 66.68	4446.22	8.30	9.80	8.90	± 3.46	11.97
Nitrate (mg/L)	83.00	94.80	88.04	± 62.18	3866.35	84.60	94.40	84.83	± 59.91	3589.21
Ammonia (mg/L)	10.80	12.00	11.22	± 7.58	57.46	2.30	3.20	2.70	± 1.87	3.50
Sulphate (mg/L)	830.60	834.60	832.43	± 411.84	169,612.19	726.60	731.60	729.69	± 500.41	250,410.17
Chloride (mg/L)	958.70	969.40	962.44	± 3.76	14.14	860.40	873.40	865.45	± 602.77	363,331.67
Calcium (mg/L)	246.70	251.80	250.17	± 106.19	11,276.32	247.00	284.80	248.44	± 150.22	22,566.05

### Results of different WQIs

In this study, several commonly used WQI models are applied by using the physicochemical parameters of the Jamuna River. The following WQI results are explained according to Bangladesh’s EQS^56^ and DoE^57^ standard guidelines.

#### WAWQI

**Figure 2:**
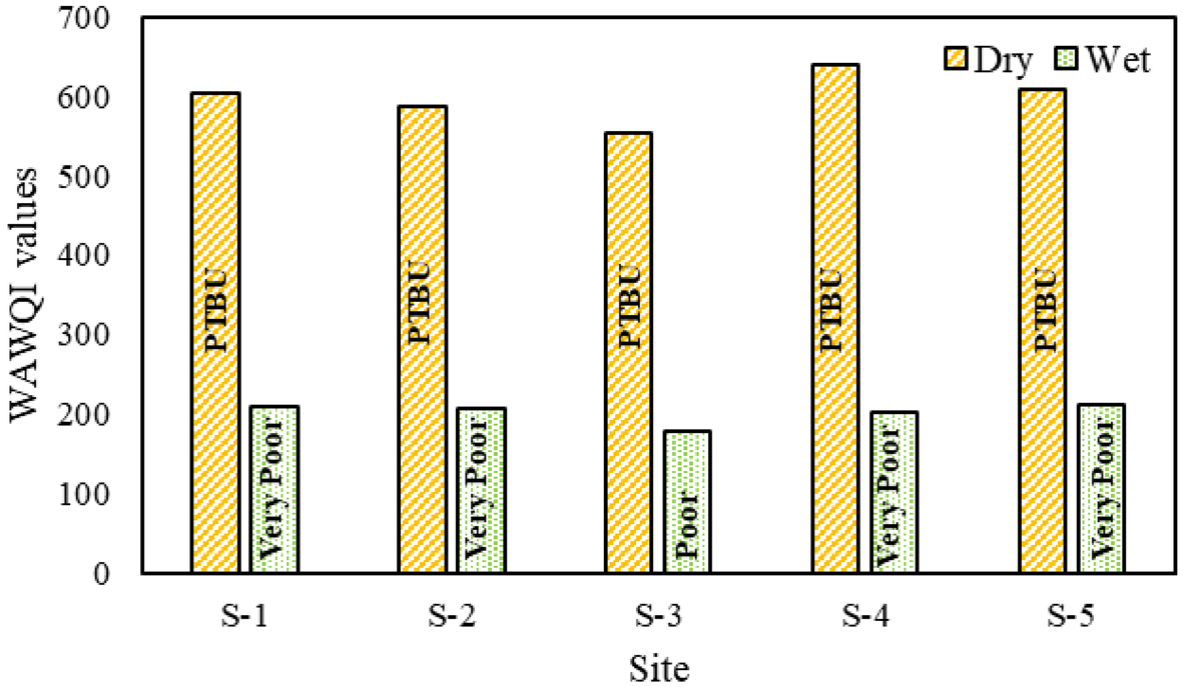
WAWQI of Jamuna River.

The results of the WAWQI for the Jamuna River at five different sites during the dry and wet seasons are presented in Fig. 2. During the dry season, site 4 exhibited the highest WAWQI value (639.21), indicating a rating of "PTBU". This site is located between the river's chars and is heavily impacted by urbanization and industrialization. Conversely, site 3 displayed the lowest WAWQI value (553.95), also classified as "PTBU". In the wet season, site 3 had the lowest WAWQI value (178.43), indicating "Poor" water quality. On the other hand, site 5 had the highest WAWQI value (212.84), classified as "Very Poor" quality. Among the water parameters, only TSS and TDS fell within the standard permissible range, while all other parameters exceeded the limits. Turbidity, ammonia, and dissolved oxygen (DO) levels were identified as primary factors contributing to the poor water quality based on the q_i_w_i_ value of WAWQI. The presence of multiple pollutants from various sources, such as surface runoff, animal and human feces, drainage system discharge, household waste, local market activities, rice mill, cement mill, and sugar mill effluents, as well as other industrial pollutants, indicates a high level of pollution in the river. Consequently, the water quality in the Jamuna River during both the wet and dry seasons is deemed unsuitable for fisheries and other household uses.

#### BCWQI

The BCWQI is a method used to evaluate the quality of a river or watershed management system in a concise manner. It aims to simplify the complex information about the ecosystem by representing it with a well-classified value. In order to calculate the WQI of the Jamuna River during the dry and winter seasons, Table 2 provides a detailed illustration. The table includes objectives for ten selected physicochemical parameters and assesses the deviation and exceedance of water quality from the EQS^56^ and DoE^57^ guidelines. While the TSS and TDS parameters have not exceeded the standard permissible value, all other parameters have. The maximum exceedance and deviation are observed in the nitrate (mg/L) values of sites 2 and 4, with a value of 99.89% during the dry season. Similarly, in the wet season, sites 2, 4, and 5 also have a value of 99.89%. These results indicate that the discharge of effluents or pollution of water by nitrate is greater during the wet season. The parameter with the least exceedance is the pH value of the Jamuna River, with a value of 1.16%, and site 3 falls within the standard limit during the dry season (pH: 8.4). Based on the classification provided in Table A4 (in Supplementary Materials), the BCWQI of the Jamuna River falls into the "Poor" water quality category, with an index value of 97.28, which is the lowest category. However, it is important to note that there are limitations in using the BCWQI to assess the water quality of a river or watershed, as mentioned by Zandbergen and Hall^22^. Therefore, in order to protect the aquatic resources, the limitations of the index should be taken into consideration when evaluating the results.

**Table 2:** BCWQI computations.

	Objectives	Dry season					Wet season				
		1	2	3	4	5	1	2	3	4	5
DO (mg/L)	> 5	0.96	1.06	1.1	0.8	1.1	0.3	0.6	0.3	0.4	0.8
Objective exceeded?		Yes	Yes	Yes	Yes	Yes	Yes	Yes	Yes	Yes	Yes
Deviation (%)		80.8	78.8	78	84	78	94	88	94	92	84
pH	6.5–8.5	8.8	8.7	8.4	8.6	8.8	9.1	8.9	8.6	8.9	9
Objective exceeded?		Yes	Yes	No	Yes	Yes	Yes	Yes	Yes	Yes	Yes
Deviation (%)		3.41	2.30	–	1.16	3.41	6.59	4.49	1.16	4.49	5.56
Turbidity (NTU)	< 10	181.82	157.77	118.53	172.96	164.10	125.59	105.83	87.22	68.62	79.08
Objective exceeded?		Yes	Yes	Yes	Yes	Yes	Yes	Yes	Yes	Yes	Yes
Deviation (%)		94.50	93.66	91.56	94.22	93.91	92.04	90.55	88.53	85.43	87.35
TSS (mg/L)	< 150	148	129	98	141	134	118	101	85	69	78
Objective exceeded?		No	No	No	No	No	No	No	No	No	No
Deviation (%)		–	–	–	–	–	–	–	–	–	–
TDS (mg/L)	< 2100	130	129.6	130.6	130.4	129.3	109	109.8	109.6	110	109
Objective exceeded?		No	No	No	No	No	No	No	No	No	No
Deviation (%)		–	–	–	–	–	–	–	–	–	–
Ammonia (mg/L)	< 1.2	11	11	10.8	12	11.38	2.4	2.7	2.3	3.1	3.2
Objective exceeded?		Yes	Yes	Yes	Yes	Yes	Yes	Yes	Yes	Yes	Yes
Deviation (%)		89.09	89.09	88.89	90.00	89.46	50.00	55.56	47.83	61.29	62.50
Nitrate (mg/L)	< 0.1	86.2	89.6	83	94.8	85.7	86.6	87.6	84.6	94.4	91.3
Objective exceeded?		Yes	Yes	Yes	Yes	Yes	Yes	Yes	Yes	Yes	Yes
Deviation (%)		99.88	**99.89**	99.88	**99.89**	99.88	99.88	**99.89**	99.88	**99.89**	**99.89**
Sulphate (mg/L)	< 22	833	831.6	834.6	830.6	832.3	730.4	731.6	730.6	726.6	729
Objective exceeded?		Yes	Yes	Yes	Yes	Yes	Yes	Yes	Yes	Yes	Yes
Deviation (%)		97.36	97.35	97.36	97.35	97.36	96.99	96.99	96.99	96.97	96.98
Chloride (mg/L)	< 13	960.4	969.4	961.8	960.4	958.7	860.4	873.4	865.8	862.4	865.3
Objective exceeded?		Yes	Yes	Yes	Yes	Yes	Yes	Yes	Yes	Yes	Yes
Deviation (%)		98.65	98.66	98.65	98.65	98.64	98.49	98.51	98.50	98.49	98.50
Calcium (mg/L)	< 36	250	251.08	250.4	250.6	246.7	247.8	284.8	249.4	249.2	247
Objective exceeded?		Yes	Yes	Yes	Yes	Yes	Yes	Yes	Yes	Yes	Yes
Deviation (%)		85.60	85.66	85.62	85.63	85.41	85.47	87.36	85.57	85.55	85.43
F1 = 80%, F2 = 79%, F3 = 99.89%									BCWQI	97.28	Poor

Significant values are in bold.

#### CWQI

The CWQ is a highly effective tool for monitoring environmental trends and safeguarding vulnerable species in the environment^32,58,59^. It is a site-specific WQI that provides valuable information about water quality at specific locations, benefiting the general public, stakeholders, and policymakers. Table 3 displays the CWQI computation values for the Jamuna River, presenting thirteen water quality parameters compared to standard objective values. All parameters, except TSS (Total Suspended Solids), TDS (Total Dissolved Solids), COD (Chemical Oxygen Demand), and one pH value, exceeded the permitted standard values (denoted in bold). During the dry season, the highest parameter values that exceeded the objective was turbidity: 181.8 NTU (S-1), followed by pH: 8.8 (S-5), ammonia: 12 mg/L (S-4), nitrate: 94.8 mg/L (S-4), sulphate: 834.6 mg/L (S-3), chloride: 969.4 mg/L (S-2), calcium: 250.6 mg/L (S-4), and BOD5 (Biochemical Oxygen Demand): 40 mg/L (S-5). In contrast, during the wet season, turbidity: 125.6 NTU (S-1), pH: 9.1 (S-1), ammonia: 3.2 mg/L (S-5), nitrate: 94.4 mg/L (S-4), sulphate: 731.6 mg/L (S-2), chloride: 873.4 mg/L (S-2), calcium: 284.8 mg/L (S-2), and BOD5: 56 mg/L (S-5) were the parameters that exceeded the objective values. The assessment reveals that site 4 experiences severe pollution from ammonia, nitrate, and calcium during the dry season. Furthermore, in the winter season, site 2 is severely contaminated by sulphate, chloride, and calcium. In both seasons, site 5 consistently surpasses the standard objective limit for the BOD5 parameter, significantly impacting fisheries. The CWQI calculation values for the Jamuna River, specifically F_1_ (scope), F_2_ (frequency), and F_3_ (amplitude), are 76.92%, 76.15%, and 85.96%, respectively. Based on Table A1 (in Supplementary Materials), following the EQS guidelines of 1997 and DoE guidelines of 2001, the CWQI results for the Jamuna River classify its water quality as "Poor" (20.2). This classification indicates that water quality in the Jamuna River is consistently at risk or degraded, deviating from natural or optimal levels^23,58^.

**Table 3:** CWQI computations.

Season	Site	DO (mg/L)	pH	Turbidity NTU	TSS (mg/L)	TDS (mg/L)	Ammonia (mg/L)	Nitrate (mg/L)	Sulphate (mg/L)	Chloride (mg/L)	Calcium (mg/L)	COD (mg/L)	BOD_5_ (mg/L)	Temperature (℃)
Dry	1	0.96	8.8	181.8	148	130	11	86.2	833	960.4	250	97	33	32.5
	2	1.06	8.7	157.8	129	129.6	11	89.6	831.6	969.4	251.08	101.6	33.8	32.5
	3	1.1	8.4	118.5	98	130.6	10.8	83	834.6	961.8	250.4	94.8	32.4	32.5
	4	0.8	8.6	173.0	141	130.4	12	94.8	830.6	960.4	250.6	98.8	34.4	32.7
	5	1.1	8.8	164.1	134	129.3	11.38	85.7	832.3	958.7	246.7	100	40	32.8
Wet	1	0.3	9.1	125.6	118	109	2.4	86.6	730.4	860.4	247.8	9.8	52.6	35.8
	2	0.6	8.9	105.8	101	109.8	2.7	87.6	731.6	873.4	284.8	8.6	49	35.9
	3	0.3	8.6	87.2	85	109.6	2.3	84.6	730.6	865.8	249.4	9.2	50.8	35.9
	4	0.4	8.9	68.6	69	110	3.1	94.4	726.6	862.4	249.2	8.8	54.4	35.9
	5	0.8	9	79.1	78	109	3.2	91.3	729	865.3	247	8.3	56	36.1
Standard (objective)		5	6.5–8.5	10.0	150	2100	1.2	10	22	600	36	200	6	25
F1 = 76.92%, F2 = 76.15% and F3 = 85.96%												CCME WQI	20.2	Poor

Bolded values mean exceedance from standard.

#### AWQI

**Figure 3:**
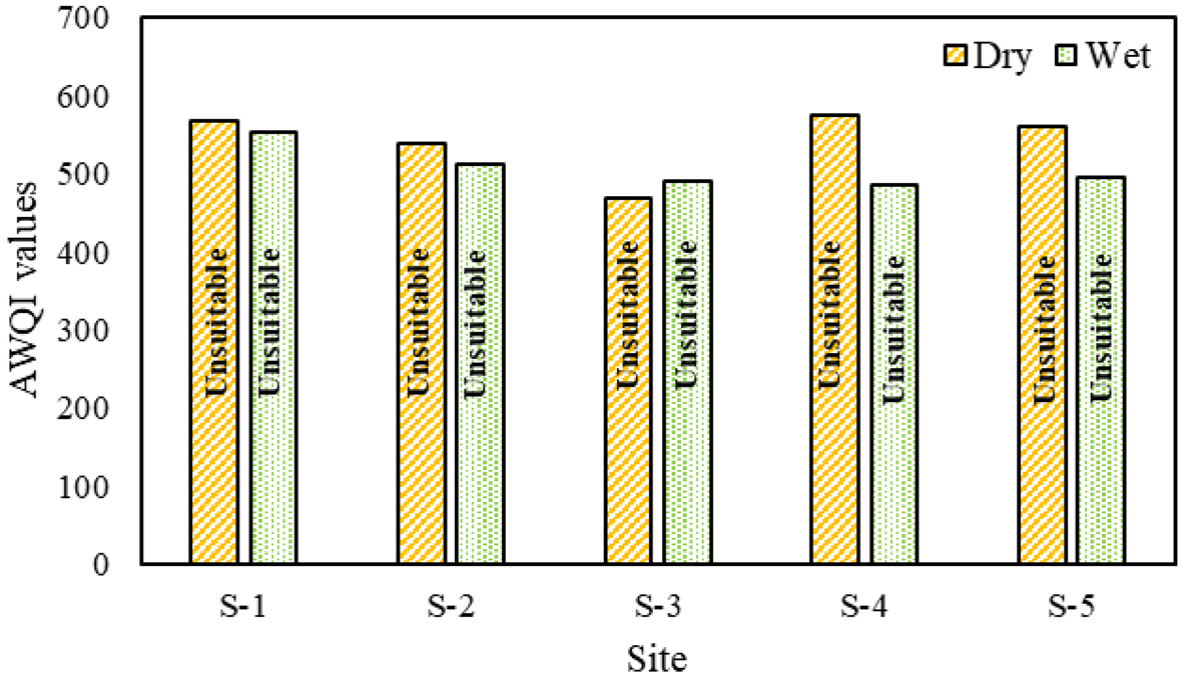
AWQI of Jamuna River.

Figure 3 illustrates the AWQI values for five locations along the Jamuna River. The data reveals that during both the winter and dry seasons, the water quality at all five sites is classified as "Unsuitable." This classification aligns with the findings of Alobaidy et al.^24^ and Ramakrishnaiah et al.^34^. Alobaidy et al.^24^ identified Dissolved Oxygen (DO) as the most pivotal parameter for water quality assessment and designated the Average Weight (AW) of DO as the maximum value. In the dry season, site 4 registers the highest AWQI value at 573.49, while site 3 records the lowest at 468.61. This suggests that water from site 4 is not fit for drinking or household use. In contrast, during the wet season, site 1 has the peak AWQI value of 553.43, with site 4 at the lowest with 485.11. This indicates that during the wet season, water from site 1 is more restricted for domestic use. The sub-indices (Sl_i_) calculations highlight that turbidity, BOD_5_ (Biochemical Oxygen Demand), and nitrate are significant contributors to the AWQI values. For instance, during the dry season, site 1, with its elevated turbidity, results in an Sl_i_ value of 266.08 out of a total AWQI of 568.33. Similarly, BOD_5_ and nitrate contribute Sl_i_ values of approximately 100.61 and 115.63, respectively. Recent observations indicate that the Jamuna River is undergoing significant pollution challenges due to rapid industrialization and climate change. As a result, the AWQI evaluations categorize its water quality as "Unsuitable."

#### MWQI

**Figure 4:**
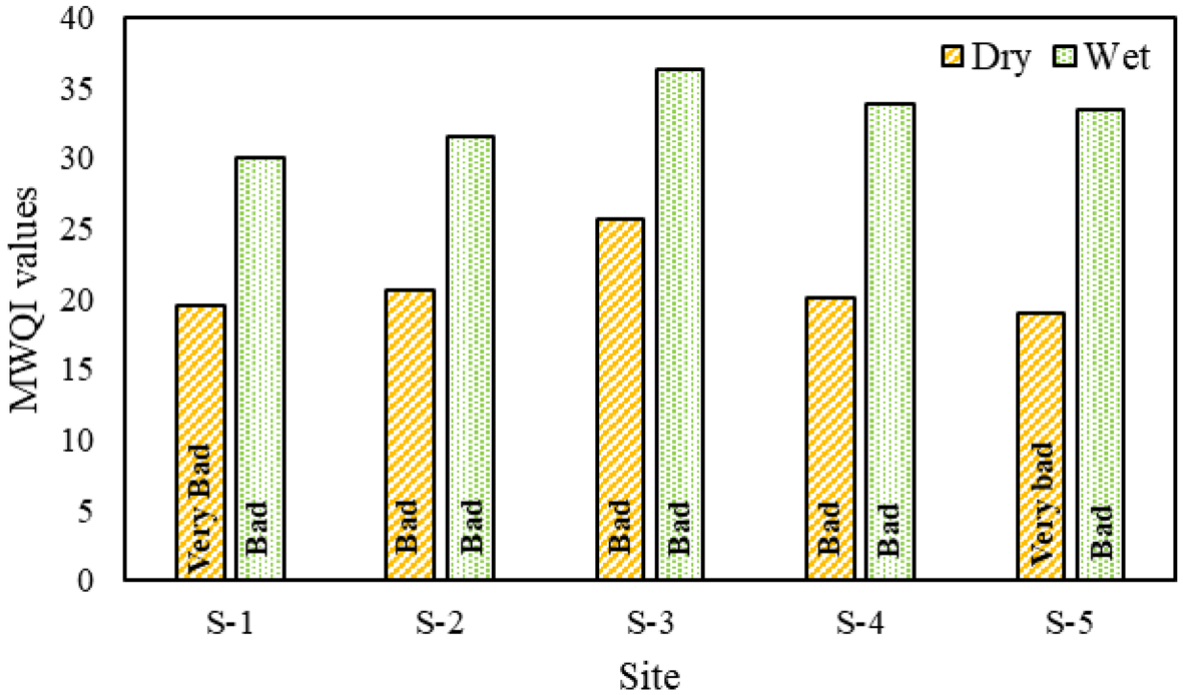
MWQI of Jamuna River.

The Department of Environment, Malaysia has developed the MWQI (Malaysia Water Quality Index) as a means of assessing the quality of river water and implementing measures to protect the aquatic environment^25^. In this study, the MWQI is presented in Fig. 4, with reference to the standard permissible value of the EQS from 1997 and the guidelines set by the Department of Environment (DoE) in Bangladesh in 2001. The MWQI is a numerical value that indicates the quality of water, with higher values representing better water quality and lower values indicating poorer water quality. During the dry season, site 1 has the lowest MWQI value of 19.57, which categorizes the water quality as "Very Bad" according to the classification in Table A8 (in Supplementary Materials). Similarly, site 3 has the highest MWQI value of 25.62, indicating "Bad" water quality. In the wet season, site 3 once again has the highest MWQI value of 36.35, also categorized as "Bad" water quality, while site 1 has the lowest value of 30.10, also indicating "Bad" water quality. Therefore, the MWQI values demonstrate that water quality is generally better in the wet season compared to the dry season at these five sites. Furthermore, when calculating the sub-indices (SI) of the MWQI, it is found that the pH sub-index has the highest value of 72.13, followed by the TSS (Total Suspended Solids) sub-index with a value of 26.60. These two factors, pH and TSS, are crucial determinants in the MWQI. Overall, the results of the MWQI indicate that the water quality in the Jamuna River is classified as "Bad" and "Very Bad" depending on the season, and it falls under Class IV according to the Malaysian water quality standards. This classification suggests that the water is only suitable for irrigation purposes^25^.

#### OWQI

**Figure 5:**
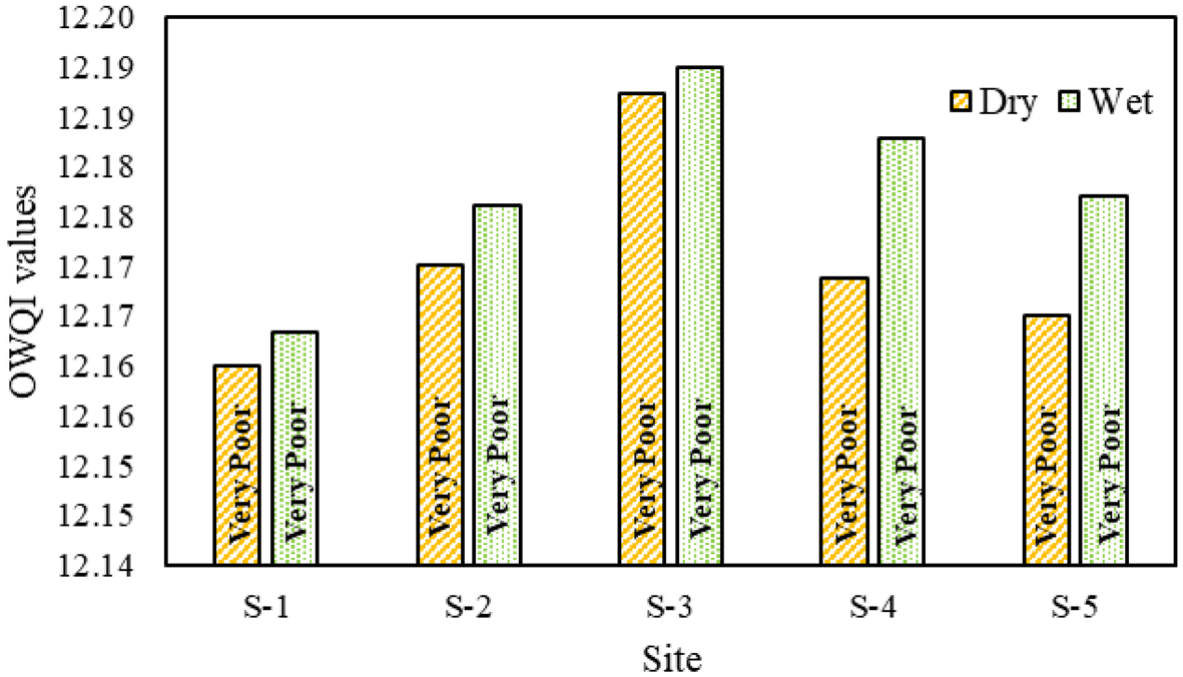
OWQI of Jamuna River.

The analysis results of the OWQI are presented in Fig. 5, which clearly identifies the five sites along the Jamuna River as having a water quality that is categorized as "Very Poor". It is worth noting that Oregon State in the United States continues to utilize the OWQI for the purpose of evaluating the quality of surface water and safeguarding the environment, as stated by the Department of Environment (DOE)^60^. The OWQI findings reveal that during the dry seasons, site 3 exhibits the highest value of 12.19, while site 1 has the lowest value of 12.16, both falling under the category of "Very Poor" quality, as indicated in Table A9 (in Supplementary Materials). Similarly, in the wet season, sites 3 and 1 maintain the same values as observed during the dry season. Furthermore, when considering the sub-indices (SI) values of OWQI at Site 1, it becomes evident that the pH value of 66.03 and the TS value of 53.6 are the primary factors influencing the calculation of the index. Based on the OWQI results, it is clear that the overall quality of the Jamuna River is deemed "Very Poor". Consequently, it is crucial to take necessary measures to mitigate the negative impact on the river and improve its water quality.

### Comparison of different WQIs

**Figure 6:**
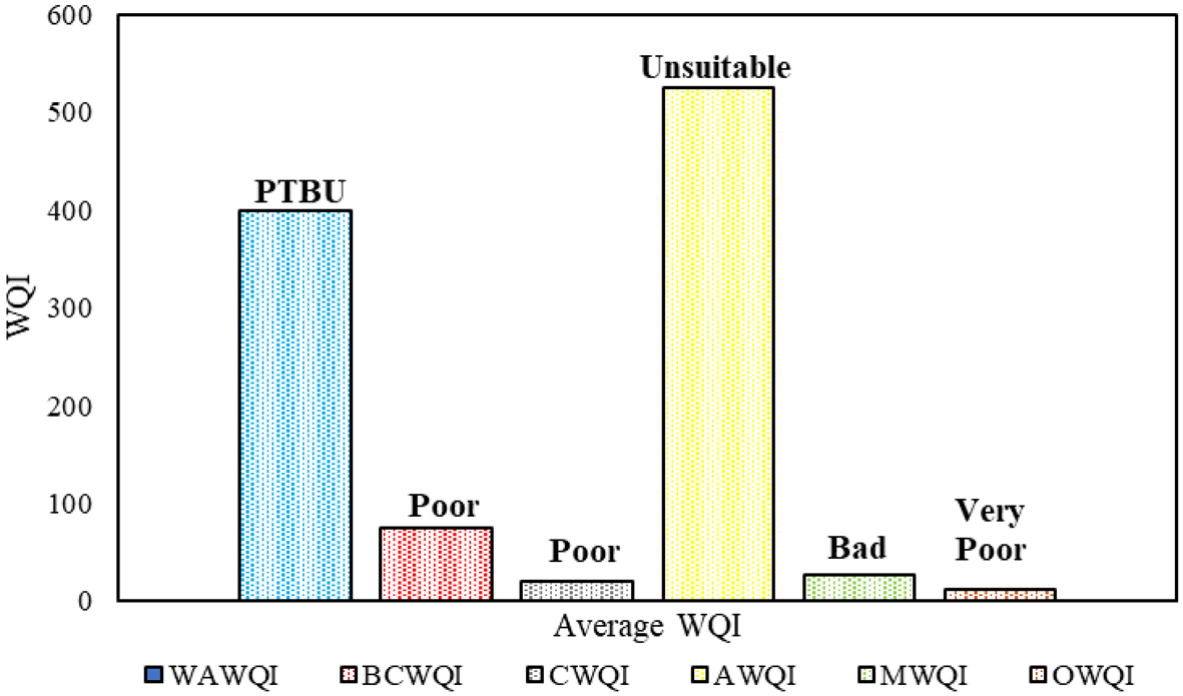
Global Six WQIs Comparison of Jamuna River.

This research assesses the water quality of the Jamuna River using multiple WQIs. These indices, as depicted in Fig. 6, have different classifications and value ranges. Some indices rely on predetermined values based on expert judgment, while others utilize unique algorithms. Furthermore, certain rating systems assess the entire river's water as a single rating, while others adopt a site-specific approach. By applying these indices to the dataset of the Jamuna River, it was observed that the water quality is inadequate for household use and is classified as low quality according to different indices. It is important to note that there are variations in the calculation and categorization of WQIs across different countries. Specifically, the CWQI and BCWQI indicate that the water quality is "Poor," while the average value of all sites according to MWQI deems it as "Bad." Additionally, the average value of all sites according to AWQI categorizes the water as "Unsuitable," and the average value of all sites according to WAWQI terms it as "PTBU." Lastly, the OWQI, based on the average value of all sites, labels the water as "Very Poor." Furthermore, during the dry season, the WAWQI and AWQI identify site 4 as having the highest WQI values, while site 3 has the lowest values, suggesting a potential relationship between these two indices. On the other hand, in both seasons, the MWQI and OWQI consistently identify site 3 as having the highest WQI values, indicating a strong correlation between these indices. Overall, the water quality of the Jamuna River is assessed as poor across all indices, indicating that it is unsuitable for the habitat of aquatic species and unfit for human consumption.

### Comparison with prior studies

Table 4 presents a compilation of previous studies conducted on the application of WQI to the rivers of Bangladesh. Various rivers in Bangladesh have been subjected to different WQI assessments, with the CWQI and WAWQI indices being the most commonly utilized. Recent studies by Mukut et al.^61^, Chowdhury et al.^62^, and Hasan et al.^63^ focused on the Karnaphuli, Shitalakshya, and Dhaleshwari rivers, respectively. The results of these studies indicate that the CWQI index consistently classifies the water quality of these rivers as "Poor." Additionally, the WAWQI results suggest that pretreatment is necessary before using the water, hence classifying it as "PTBU." Furthermore, Muyen et al.^64^ applied the MWQI to the historic Brahmaputra River, revealing that the water quality is categorized as "Very Polluted," indicating the lowest level of water quality. Moreover, Mallick et al.^65^ evaluated eleven rivers in Bangladesh using WQI, considering only the pH, DO, BOD, and SS parameters. The outcomes of the WQI assessments revealed that the majority of these rivers are polluted and fall into the lowest water quality category. The authors of these studies consistently attribute industrial effluent, anthropogenic activities, municipal wastewater, and runoff as the primary sources of pollution. Additionally, the studies highlight that pollution is more prevalent during the dry season compared to the wet season. Consequently, these findings emphasize the urgent need for precautions and measures to address the poor water quality of these rivers, which are vital sources of water for the local population. To effectively measure the WQI of surface water quality in Bangladesh, given its significance as a water supply for the locals, it is imperative to establish the necessary norms and guidelines. Therefore, it is crucial to thoroughly assess the current state of water quality, identify the sources of pollution, and implement appropriate measures to ensure the improvement and preservation of water resources.

**Table 4:** WQI comparisons of various rivers of Bangladesh.

S. no.	River Name	District	WQI	Rank	Results	References
1	Karnaphuli River	Chittagong	CCME WQI	Poor	Industrial waste, municipal trash, runoff, etc., all contributed to the pollution. Water is unsuitable for any use	Mukut et al.,^61^
2	Shitalakshya River	Narayanganj	CCME WQI	Poor	Most pollution comes from human activities and waste products from factories. Unfit for use in agriculture, drinking, or pisciculture. The water quality throughout the winter months is far worse than during the monsoons	Chowdhury et al.,^62^
			WAWQI	PTBU		
3	Dhaleshwari River	Tangail	CCME WQI	Poor	The majority of pollution comes from industrial and municipal waste products such tannery effluent, sewage, runoff, and other types of wastewaters. The water quality throughout the winter months is far worse than during the monsoons	Hasan et al.,^63^
			WAWQI	PTBU		
4	Turag River	Gazipur	WAWQI	PTBU	The main causes of pollution include industrial effluent, sewage waste, runoff, and anthropogenic activities	Tahmina et al.,^66^
5	Old Brahmaputra River	Mymensingh	MWQI	Very Polluted	The majority of pollution originates from human activities and industrial byproducts, as well as municipal wastewater, runoff, and other similar sources. This water should only be used for irrigation	Muyen et al.,^64^
6	Jamuna River	Tangail	WAWQI	PTBU	Industrial effluents, anthropogenic activities, runoff and municipal wastewater discharge are main possible pollution reason. Proper treatment required before using the water	Present study
			BCWQI	Poor		
			CCME	Poor		
			AWQI	Unsuitable		
			MWQI	Bad		
			OWQI	Very poor		

*PTBU* proper treatment required before use.

## Conclusions

The Jamuna River is home to a fragile ecosystem, and its water quality fluctuates with the changing seasons. Unfortunately, industrialization and urbanization have significantly damaged the river's ecosystem. At five monitoring stations, it has been observed that during both dry and wet seasons, the levels of DO, pH, turbidity, ammonia, nitrate, sulphate, chloride, calcium, BOD5, COD, and temperature parameters exceed the standard permissible range set by the EQS^56^ and DoE^57^ guidelines. In order to accurately evaluate the pollution in the river's surface water, this study examines the water quality using various WQI models. These WQI methods, such as WAWQI, BCWQI, CWQI, AWQI, MWQI, and OWQI, are recognized worldwide as powerful and concise tools for assessing water pollution. The results of all six WQI methods indicate that the water quality of the Jamuna River falls into the lowest category, suggesting a strong correlation between these indices and accurate determination of surface water quality.

Due to its poor quality, the water of the Jamuna River should not be used for drinking or household purposes. The continuous contamination of the river occurs through the discharge of industrial effluents, open defecation, and runoff from urban and agricultural areas, leading to a significant degradation of water quality on a daily basis. Previous research has also shown that the majority of Bangladesh's river water quality falls into the lowest category of the WQI, indicating very poor water quality. Therefore, it is crucial to take necessary actions to improve the water quality for consumption. If the water is to be used for drinking, agriculture, or household purposes, it must undergo continuous monitoring and treatment. Additionally, the country's administration should implement necessary restoration methods to improve the health and water quality of the river.

### Supplementary Information

Supplementary Information.

## Data Availability

Further information/data may be supplied upon request. For access to the data and materials, please contact A. Ahsan at ashikcivil@yahoo.com.

## Abbreviations

WQI: Water quality index
WHO: World health organization
WAWQI: Weighted arithmetic water quality index
CCME: Canadian council of ministers of the environment
BCWQI: British Columbia water quality index
CWQI: Canadian water quality index
AWQI: Assigned water quality index
MWQI: Malaysian water quality index
OWQI: Oregon water quality index
DOE: Department of environment
NSWQI: National sanitation foundations water quality index
SI: Sub-indices
EQS: Environmental quality standards
EC: Electrical conductivity
BOD: Biological oxygen demand
TDS: Total dissolved solids
TSS: Total suspended solids
TS: Total solids
DO: Dissolved oxygen
COD: Chemical oxygen demand
FDEP: Florida Department of environmental protection
NTU: Nephelometric turbidity unit
PTBU: Proper treatment required before use
