# Supplementary material for: Evaluation of the surface water quality using global water quality index (WQI) models: perspective of river water pollution
Source: Sci Rep. 2023 Nov 22;13:20454. doi: 10.1038/s41598-023-47137-1 (PMC10665448; doi:10.1038/s41598-023-47137-1)
Supplement: Supplementary file 1 — Supplementary Information. [file 41598_2023_47137_MOESM1_ESM.docx]

**Supplementary Materials**

**Table A1: Jamuna River Dataset**

| Parameters | Standard | Dry season (December- February) | | | | | Wet season (April- September) | | | | | References of standards |
| --- | --- | --- | --- | --- | --- | --- | --- | --- | --- | --- | --- | --- |
|  |  | 1 | 2 | 3 | 4 | 5 | 1 | 2 | 3 | 4 | 5 |  |
| DO (mg/L) | >5 | 0.96 | 1.06 | 1.1 | 0.8 | 1.1 | 0.3 | 0.6 | 0.3 | 0.4 | 0.8 | EQS, 1997 (Water usable by fishes) |
| DO (in % saturation) |  | 13.26 | 14.64 | 15.19 | 11.09 | 15.27 | 4.37 | 8.76 | 4.38 | 5.84 | 11.72 |  |
| pH | 6.5-8.5 | 8.8 | 8.7 | 8.4 | 8.6 | 8.8 | 9.1 | 8.9 | 8.6 | 8.9 | 9 | EQS, 1997 (Water usable by fishes) |
| Turbidity (NTU) | 10 | 181.8 | 157.8 | 118.5 | 173.0 | 164.1 | 125.6 | 105.8 | 87.2 | 68.6 | 79.1 | EQS, 1997 (Inland surface water standard from industrial effluents) |
| TSS (mg/L) | 150 | 148 | 129 | 98 | 141 | 134 | 118 | 101 | 85 | 69 | 78 | EQS, 1997 (Inland surface water standard from industrial effluents) |
| TDS (mg/L) | 2100 | 130 | 129.6 | 130.6 | 130.4 | 129.3 | 109 | 109.8 | 109.6 | 110 | 109 | EQS, 1997 (Inland surface water standard from industrial effluents) |
| TS (mg/L) |  | 278 | 258.6 | 228.6 | 271.4 | 263.3 | 227 | 210.8 | 194.6 | 179 | 187 |  |
| Ammonia (mg/L) | 1.2 | 11 | 11 | 10.8 | 12 | 11.38 | 2.4 | 2.7 | 2.3 | 3.1 | 3.2 | Water used for pisciculture |
| Nitrate (mg/L) | 10 | 86.2 | 89.6 | 83 | 94.8 | 85.7 | 86.6 | 87.6 | 84.6 | 94.4 | 91.3 | EQS, 1997 (Inland surface water standard from industrial effluents) |
| Sulphate (mg/L) | 22 | 833 | 831.6 | 834.6 | 830.6 | 832.3 | 730.4 | 731.6 | 730.6 | 726.6 | 729 | DoE,2001 |
| Chloride (mg/L) | 600 | 960.4 | 969.4 | 961.8 | 960.4 | 958.7 | 860.4 | 873.4 | 865.8 | 862.4 | 865.3 | EQS, 1997 (Inland surface water standard from industrial effluents) |
| Calcium (mg/L) | 36 | 250 | 251.08 | 250.4 | 250.6 | 246.7 | 247.8 | 284.8 | 249.4 | 249.2 | 247 | DoE,2001 |
| COD (mg/L) | 200 | 97 | 101.6 | 94.8 | 98.8 | 100 | 9.8 | 8.6 | 9.2 | 8.8 | 8.3 | EQS, 1997 (Inland surface water standard from industrial effluents) |
| BOD_5_ (mg/L) | 6< | 33 | 33.8 | 32.4 | 34.4 | 40 | 52.6 | 49 | 50.8 | 54.4 | 56 | EQS, 1997 (water usable by fishes) |
| EC (µS/cm) | 1200 | 137.2 | 137.6 | 138.2 | 138 | 138.3 | 107 | 107 | 106.4 | 105.1 | 105 | EQS, 1997 (Inland surface water standard from industrial effluents) |
| Temperature (℃) | 25 | 32.5 | 32.5 | 32.5 | 32.7 | 32.8 | 35.8 | 35.9 | 35.9 | 35.9 | 36.1 | EQS, 1997 (Water usable by fishes) |

**Table A2: Classification of WAWQI values** (Jerome and Pius, 2010)

| Range | Water Quality |
| --- | --- |
| <50 | Excellent |
| 50-100 | Good |
| 100-200 | Poor |
| 200-300 | Very Poor |
| Above 300 | Proper treatment required Before use (PTBU) |

**Table A3: Unit weight of WAWQI**

| Parameters | Standard | 1/Si | wi |
| --- | --- | --- | --- |
| DO (ppm) | 5 | 0.2 | 0.1215 |
| DO (in % saturation) |  |  |  |
| pH | 6.5-8.5 | 0.1176 | 0.0714 |
| Turbidity NTU | 10 | 0.1 | 0.0607 |
| TSS (ppm) | 150 | 0.0066 | 0.0040 |
| TDS (ppm) | 2100 | 0.0004 | 0.0002 |
| NH3-N (mg/L as N) or Ammonia | 1.2 | 0.8333 | 0.5064 |
| Nitrate (ppm) | 10 | 0.1 | 0.0607 |
| Sulphate (ppm) | 22 | 0.0454 | 0.0276 |
| Chloride (ppm) | 600 | 0.0016 | 0.0010 |
| Calcium (ppm) | 36 | 0.0277 | 0.0168 |
| COD (ppm) | 200 | 0.005 | 0.0030 |
| BOD_5_ (ppm) | 6 | 0.1666 | 0.1012 |
| EC (µS/cm) | 1200 | 0.0008 | 0.0007 |
| Temperature (℃) | 25 | 0.04 | 0.0243 |
| Total | | 1.6455 | 1.00025321 |
| k= 0.6077 | | | |

**Table A4: Classification of BCWQI values** (Zandbergen and Hall, 1998)

| Range | Water Quality |
| --- | --- |
| 0-3 | Excellent |
| 4-7 | Good |
| 18-43 | Fair |
| 44-59 | Borderline |
| 60-100 | Poor |

**Table A5: Classification of CWQI values** (CCME, 2001)

| Range | Water Quality |
| --- | --- |
| 95-100 | Excellent |
| 80-94 | Good |
| 60-79 | Fair |
| 45-59 | Marginal |
| 0-44 | Poor |

**Table A6: Calculation of AWQI**

| Parameters | Water quality standard | Assigned weight (AW) | Relative weight (RW) |
| --- | --- | --- | --- |
| DO (mg/L) | 5.00 | 4.00 | 0.24 |
| pH | 7.50 (Avg.) | 2.10 | 0.13 |
| Turbidity NTU | 10.00 | 2.40 | 0.15 |
| Nitrate (mg/L) | 10.00 | 2.20 | 0.13 |
| BOD_5_ (mg/L) | 6.00 | 3.00 | 0.18 |
| EC (µS/cm) | 1200.00 | 2.70 | 0.16 |
| Total | | ΣAWi = 16.40 | ΣRWi = 1 |

**Table A7: Classification of AWQI values** (Ramakrishnaiah et al., 2009)

| Range | Water Quality |
| --- | --- |
| <50 | Excellent |
| 50-100 | Good |
| 100-200 | Poor |
| 200-300 | Very Poor |
| Above 300 | Unsuitable |

**Table A8: Classification of MWQI** (DOE, 2008)

| Range | Water Quality |
| --- | --- |
| 80-100 | Excellent |
| 60-80 | Good |
| 40-60 | Moderate |
| 20-40 | Bad |
| 0-20 | Very Bad |

**Table A9: Classification of OWQI** (Cude, 2001)

| Range | Water Quality |
| --- | --- |
| 10-59 | Very Poor |
| 60-79 | Poor |
| 80-84 | Fair |
| 85-89 | Good |
| 90-100 | Excellent |
